# Effects of Graphene-Based Far-Infrared Compression Garments on Aerobic Capacity in Healthy Young Males: A Randomized Crossover Trial

**DOI:** 10.1186/s40798-025-00913-x

**Published:** 2025-10-10

**Authors:** Yuxin Peng, Dongmei Wang, Haotian Zheng, Hao Qian, Chen Chen, Yi Han, Kewei Zhao, Tianhao Gao, Wenming Liu, Xiangsheng Pang

**Affiliations:** 1 Department of Sports Science, Zhejiang University, Hangzhou, Zhejiang China; 2 Beijing Sport University, Beijing, China; 3 Wuhan Sport University, Wuhan, Hubei China; 4 Research Institute of Zhejiang University, Shaoxing, Zhejiang China; 5 Xijie Thermal Management Technology Co., Ltd, Shaoxing, Zhejiang China; 6 Gaoxi Heat Dissipation Material Technology Co., Ltd, Hangzhou, Zhejiang China; 7 China Institute of Sport Science, Beijing, China

## Abstract

**Background:**

Advancements in sports materials have led to the creation of innovative fabrics aimed at enhancing athletic performance and reducing the risk of sports-related injuries. Graphene-based composite fibers, with superior far-infrared (FIR) emissivity, are emerging as a promising material in sportswear. This study explores the effects of graphene-based FIR compression garments on aerobic exercise capacity.

**Results:**

A total of 15 healthy, recreationally active male university students (aged 18–25 years) participated in this double-blind, randomized crossover trial. Each participant completed two incremental treadmill tests while wearing either graphene-based FIR compression garments or control garments, with a 7-day washout period between sessions. Results showed significantly longer exercise durations (38.4 s, *p* < 0.001) and extended time to anaerobic threshold (37.7 s, *p* < 0.001) in those wearing graphene-based FIR garments compared to those wearing control garments. The maximum heart rate was significantly lower in the graphene group (198.8 ± 7.8 bpm vs. 200.3 ± 7.5 bpm, *p* < 0.05), with reduced heart rates at the same exercise intensity (176.9 ± 8 bpm vs. 179.8 ± 7.8 bpm, *p* < 0.05). No significant differences in Maximal Oxygen Uptake (VO2max) were observed between the two groups.

**Conclusions:**

Graphene-based FIR compression garments significantly enhance aerobic performance by improving endurance, likely due to improved peripheral blood circulation and reduced cardiac load. These findings highlight the potential of graphene-based fibers as a disruptive innovation in sportswear. Further research with larger sample sizes is warranted to fully explore their benefits.

**Supplementary Information:**

The online version contains supplementary material available at 10.1186/s40798-025-00913-x.

## Key Points

Graphene-based FIR compression garments significantly improve endurance time and delay the onset of anaerobic metabolism in healthy young males.

These garments were associated with lower heart rates at equivalent exercise intensities, suggesting improved cardiovascular efficiency and reduced cardiac load during aerobic exercise.

Graphene-based FIR garments represent a promising innovation for improving aerobic performance and should be considered in future sportswear development and recovery protocols.

**Supplementary Information:**

The online version contains supplementary material available at 10.1186/s40798-025-00913-x.

## Background

With the continuous development of sports materials, various fabrics designed to enhance human athletic performance have been introduced [1, 2]. These materials not only boost athletes’ performance but also improve the conditions for the general public, reducing the risk of sports-related injuries [3]. In the pursuit of excellence in athletic performance, elite athletes and coaches share a common goal: to push the limits of human potential and break world records. Whether it is the brief success of the “sharkskin” swimsuit or Kipchoge’s challenge of breaking the 2-hour marathon barrier, these events underscore humanity’s keen interest in advancing science to explore the boundaries of physical capability [4, 5]. Furthermore, the technological iteration of sports equipment has not only provided athletes with superior training environments but has also enhanced the sports experience for ordinary individuals, enabling them to effectively prevent injuries and achieve better performance outcomes.

Far Infrared Radiation (FIR) is a specific wavelength band within the infrared spectrum of electromagnetic radiation (3 μm to 1 mm) [6]. Most researchers believe that the optimal FIR wavelength range for producing biological effects in the human body is between 4 and 14 μm [7, 8], as it can penetrate human skin and tissues up to 4 cm [6], thereby promoting blood circulation and tissue oxygenation [9–11]. Graphene’s superior FIR emissivity matches human tissue absorption spectra, enhancing tissue temperature, blood flow, and glucose uptake via AMPK and GLUT4 activation, thereby improving endurance. Additionally, in microbiota dysbiosis models, graphene FIR devices improved exercise capacity and metabolic function [12]. Notably, Yu et al. demonstrated that graphene-based FIR flexible devices reduced tumor growth by 42% and metastatic lung nodules by 55%, underscoring the broad bioactive potential of graphene FIR applications [13].Consequently, FIR has garnered significant attention over time, particularly in its application in medical and physical therapies for treating and alleviating various conditions or aiding in athlete recovery after exertion [14, 15]. In recent years, scholars have continually explored the effects of wearing FIR-emitting garments before [16], during [17], and after exercise [18], investigating their potential to enhance athletic performance or facilitate recovery [19]. FIR works by promoting the generation of Nitric Oxide (NO) [9], which directly influences vasodilation and reduces vascular resistance. Additionally, it enhances local blood flow and tissue perfusion, thereby improving oxygen transport capacity [6, 7]. FIR can stimulate the activity of Cytochrome C Oxidase (CCO), a key enzyme in the mitochondrial respiratory chain, by photon absorption, thereby promoting ATP synthesis and enhancing mitochondrial respiration, which ultimately supports prolonged exercise endurance and reduces fatigue [20, 21], a mechanism crucial for extending exercise duration and reducing fatigue.

However, current FIR garments are typically composed of bioceramic fibers or powders embedded in fabric or bioceramic sheets/patches attached to the fabric [22–25]. These bioceramic materials are made of various metal oxides, such as magnesium, silicon dioxide, aluminum, and tourmaline. Nevertheless, their lack of stability limits the exploration of their use in enhancing athletic performance during exercise. To overcome this limitation, a new FIR material—graphene-based composite fiber—has emerged in recent years, which is expected to become the “land-based sharkskin.” In 2011, Gao and Xu first developed a new carbon fiber—graphene fiber—by using wet spinning technology to transform liquid crystal graphene oxide into fibers. This fiber has the potential for mass production, possesses a liquid crystal structure spontaneously formed by graphene oxide, self-fusion, and self-healing capabilities, as well as various low-cost reduction methods [26]. Graphene materials, due to their far-infrared spectrum overlapping with the human body’s spectrum and their ability to release negative ions, have become a focal point in FIR material research. In 2019, Hangzhou GaoxiTech Co., Ltd. invented a multifunctional nano-composite fiber based on graphene material produced through in-situ polymerization. Compared to traditional composite fibers produced by mixing polymer solutions with nano-filler solutions, this graphene-based composite fiber has unparalleled material advantages, including higher tensile strength and elastic modulus, with 92% infrared emissivity and a negative ion release rate of 5920 cm^− 3^. Therefore, graphene composite fibers, known as the fourth generation of fibers [27], have become the focus of current material research.

In this study, we used this graphene composite fiber as the textile material to create FIR compression garments, aiming to overcome the limitations of current FIR materials and explore their potential to enhance aerobic exercise capacity. The purpose of this study is to evaluate the effects of this graphene fiber-based clothing on the aerobic exercise capacity of the general population through incremental treadmill tests. We aim to elucidate the physiological mechanisms by which FIR clothing improves aerobic exercise capacity by monitoring changes in heart rate and respiratory metabolism indices during rest and exercise.

## Methods

This is a double-blind, randomized crossover controlled study aimed at evaluating the performance of physically active subjects under controlled conditions during incremental load exercise. During the experiment, subjects wore either FIR fabric compression garments or conventional material compression garments. The study adhered to the Good Clinical Practice guidelines of the Helsinki Declaration and was conducted according to the protocol approved by the Ethics Committee of Zhejiang University, approval number [2023] 067.

### Participants

Participants were recruited via university mailing lists and on-campus posters. All participants were students from Zhejiang University and received a cash incentive upon completion of the study. To reduce variability caused by hormonal fluctuations affecting cardiovascular and thermoregulatory responses during exercise, only male participants were included in this study. Participants were selected based on the following inclusion criteria: (1) male university students aged 18–25, (2) no history of cardiovascular, respiratory, or musculoskeletal disorders, (3) regularly physically active (at least 4 h/week), and (4) not taking medications affecting cardiovascular function. All participants provided informed consent and were screened through a health questionnaire. A total of 20 male university students were recruited and screened for this experiment. However, 1 participant withdrew due to illness, 2 participants were unable to continue and opted out of the experiment, and 2 others withdrew for personal reasons. Consequently, 15 participants completed the study. These 15 male students, aged 18–22 years (± 1.2), had body weights ranging from 53 to 82 kg (± 7.7), heights from 163 to 186 cm (± 5.6), and BMIs between 18.04 and 26.17 (± 2.1). All participants regularly engaged in various physical activities (6.1 ± 2.1 h per week) such as running, tennis, basketball, and soccer, and had no history or clinical symptoms of cardiopulmonary or orthopedic diseases. Each participant was fully informed of the experimental procedures and their rights and provided written informed consent. The sample size in this study was determined based on prior research employing crossover designs in physiological assessments of wearable technology. A power analysis using G*Power 3.1 was performed based on pilot data (effect size d = 0.8 for endurance time difference), with an alpha of 0.05 and a power (1-β) of 0.8. This analysis indicated a required sample size of 12 participants. To account for potential dropouts, 20 participants were initially recruited.

### Design

**Fig. 1:**
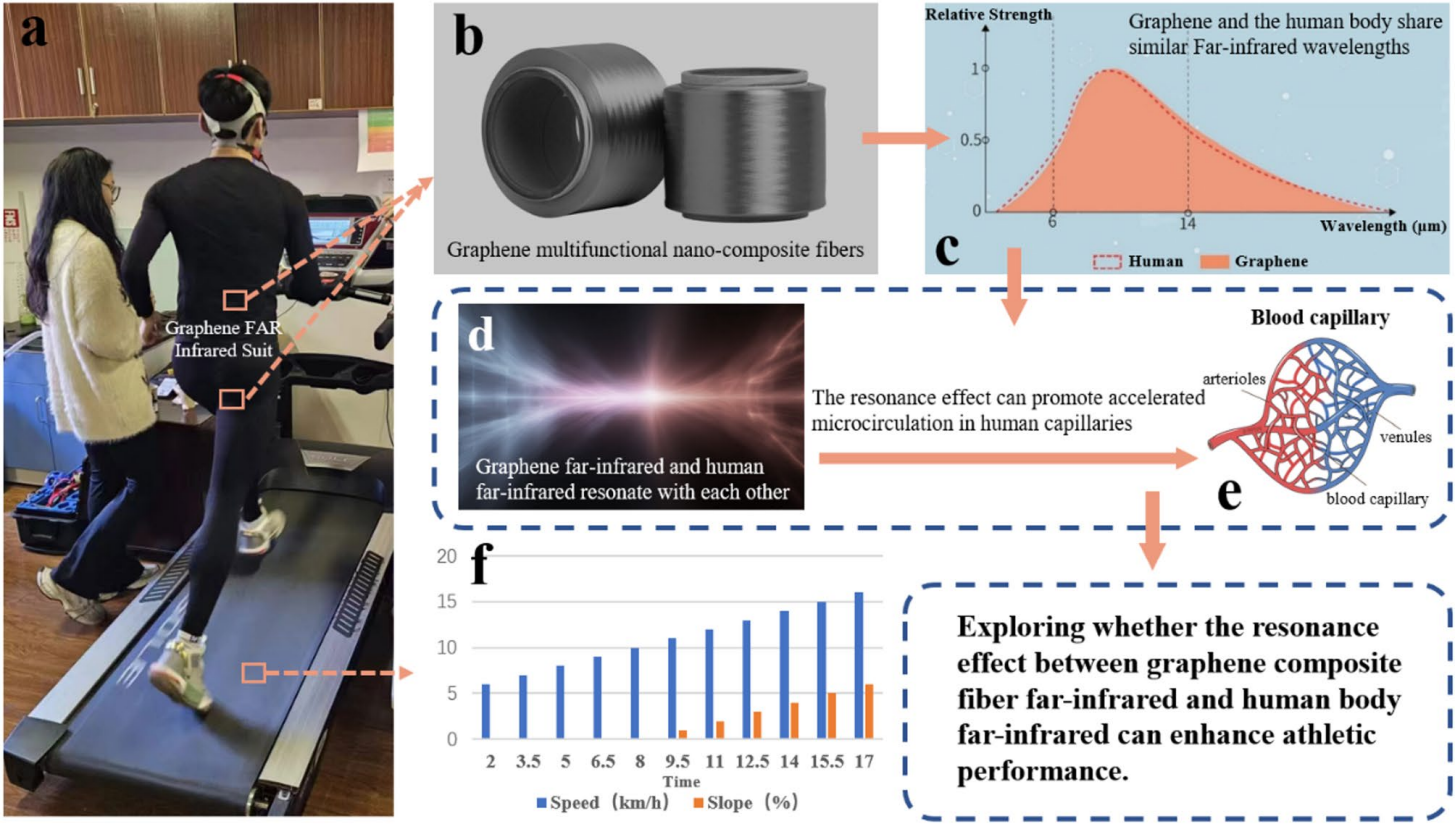
Investigating the impact of resonance between graphene composite fibers and human far-infrared radiation on athletic performance via incremental load testing

Participants were randomly assigned to one of two sequences using a computer-generated randomization list: Group A wore graphene FIR garments in the first test and control garments in the second, while Group B did the reverse. A 7-day washout period between tests was implemented to minimize carryover and adaptation effects. To further reduce bias, both participants and experimenters were blinded to garment assignments. All subjects performed two incremental load treadmill tests to measure maximal oxygen uptake (VO2 max) under identical environmental conditions (laboratory temperature of 22 °C and relative humidity of 50%) using a treadmill (h/p/cosmos, Nussdorf-Traunstein, Germany). The tests were conducted one week apart, with subjects wearing either FIR garments or control garments. Throughout the study, subjects were instructed not to alter their dietary habits or physical activity levels. Specifically, they were advised to avoid caffeine and alcohol intake and refrain from intense exercise the day before each test (Fig. 1).

The incremental load treadmill protocol, as shown in Table 1, was used for the VO2 max tests. Subjects wore a cardiopulmonary metabolic system (Cosmed, Rome, Italy) and sat quietly for 5 min to collect baseline data before starting the treadmill exercise. The Cosmed system collected real-time respiratory metabolic data, and heart rate (HR) was continuously monitored throughout the test. Subjects continued until they reached voluntary exhaustion.

**Table 1:**
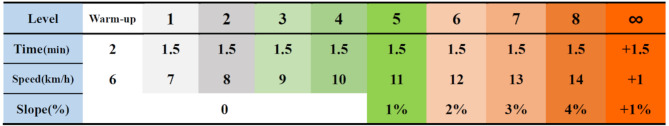
Incremental treadmill test protocol: 6 km/h warm-up 2 min, speed every 1. 5 min plus 1 km/h, each speed lasting 1.5 min, 10 km/h every 1.5 min to increase the slope to 1%, 2%, 3%, 4%, 5% exercise to exhaustion

Abbreviations: km·h⁻¹, Kilometers per hour; min, minutes; ∞, infinite

Peak VO2 was measured at the assumed maximum effort, averaged over 20 s. VO2 max was considered achieved if the following criteria were met: a stable VO2 despite increased workload, a respiratory exchange ratio (RER) of 1.1 or greater, and an HR of 85% or greater of the predicted maximum. Peak VO2 was expressed in absolute terms (ml/kg/min) and as a percentage of the predicted maximum (VO2%).

Additionally, the anaerobic threshold (AT) was determined through the V-slope analysis of carbon dioxide output (VCO2) versus VO2 curves and verified by subsequent behavior of the end-tidal partial pressures of carbon dioxide and oxygen and ventilation. O2 pulse at AT (AT O2 pulse), AT time, and AT time as a percentage of total endurance time (AT/ET ratio) were reported. VCO2 at AT (AT VCO2), HR at AT (AT HR), and VO2 at AT (AT VO2) were expressed in absolute terms (ml/kg/min) and as a percentage of predicted VO2 max (AT VO2%). As this study involved a crossover design, each participant served as their own control. We did not impose any structured training program but instead required participants to maintain their regular physical activity routines. Participants were instructed to avoid changes in activity type, intensity, frequency, or volume, and were asked to refrain from intense exercise 24 h before each trial. Compliance was verified via daily exercise logs.

## Material

**Fig. 2:**
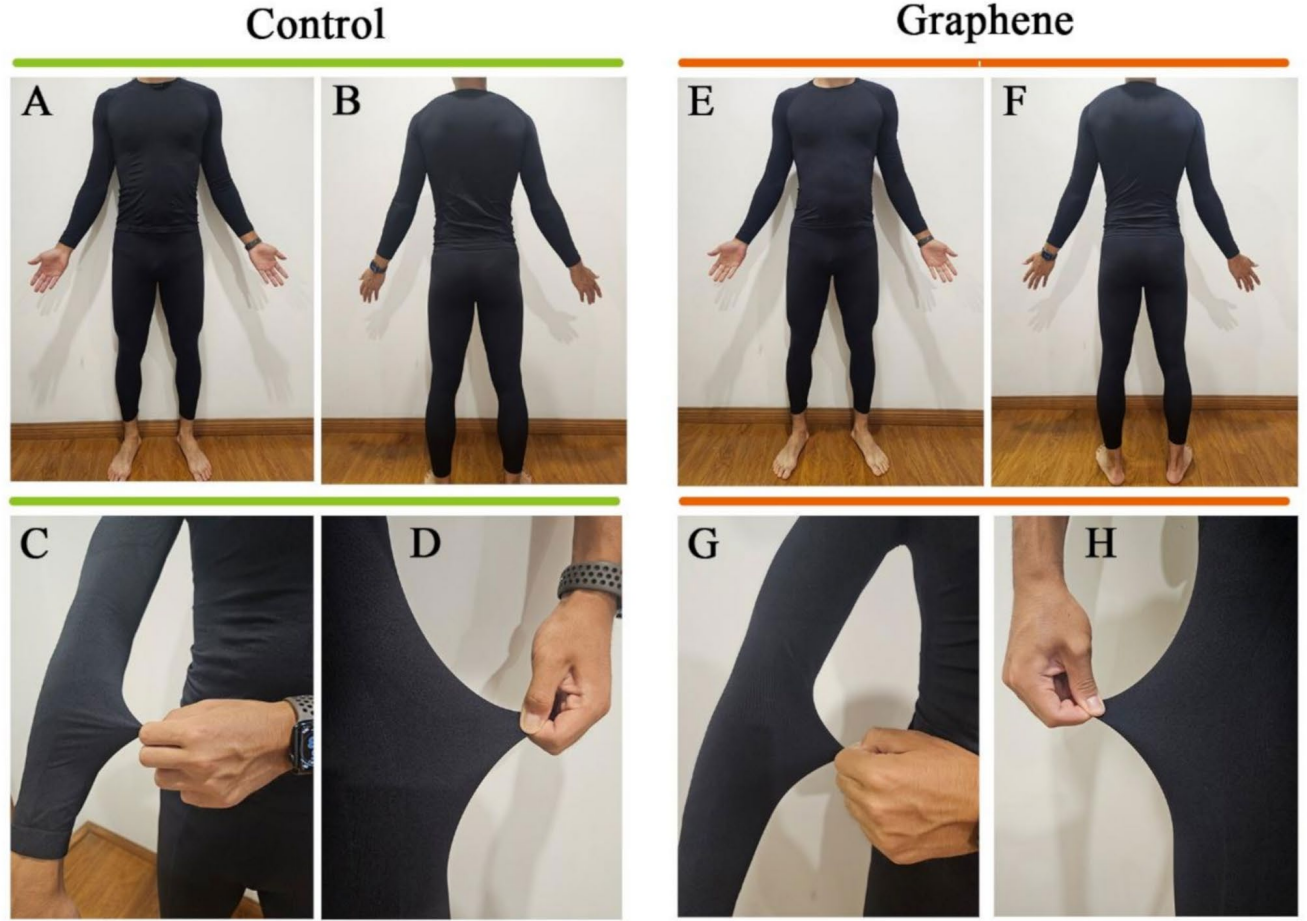
Comparison of garments between control (**A–D**) and graphene groups (**E–H**). Panels A and B (Control) and E and F (Graphene) show the overall appearance of the long-sleeved compression shirts and compression pants from front and back views, respectively. Panels C and G illustrate the tightness and elasticity of the shirt sleeves, while panels D and H demonstrate the tightness and elasticity of the compression pant legs in the control and graphene groups, respectively

To better explore the effects of graphene far-infrared (FIR) on athletic performance and its physiological mechanisms, we selected multifunctional nano-composite fibers (single-layer graphene oxide nylon 70D68F) produced by Hangzhou GaoxiTech and nylon yarns of the same specifications without graphene. The experimental compression garments were produced by Yiwu Shi’an Garment Company in Zhejiang, China. The graphene FIR garments and the control group garments included a long-sleeved compression shirt and pants, with no differences in color, structure, texture, or wearing experience (Fig. 2).

**Fig. 3:**
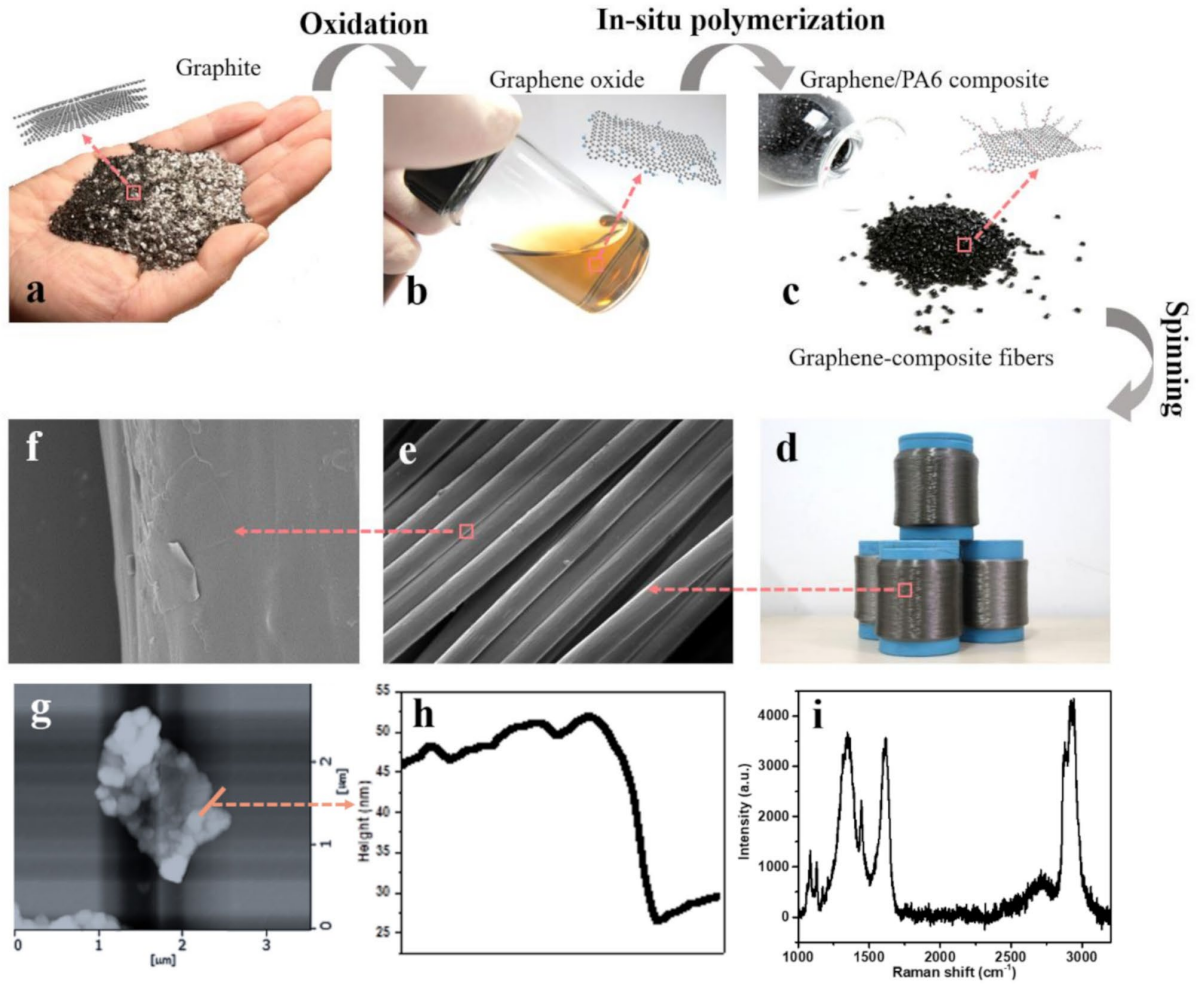
Graphite **(a)** undergoes an oxidation reaction to yield single-layer graphene oxide **(b)**. This is followed by an in-situ polymerization reaction to produce graphene composite nylon 6 (or PET) pellets **(c)**. Finally, these pellets are spun into graphene-composite fibers **(d)**. The surface morphology of the resulting fibers is observed under scanning electron microscopy **(e)**, revealing a smooth and uniform fiber surface. A higher-magnification image **(f)**. Under atomic force microscopy **(g)**, distinct step features **(h)** are observed in the height measurements, thus demonstrating the covalent bonding between graphene and the polymer. Raman characteristic peaks of graphene composite fibers **(i)**

Unlike current FIR clothing materials that embed bioceramics or precious metals in powder or fiber form into fabrics to generate far-infrared radiation, the graphene composite fibers used in this study were made by mixing graphene monomers with nano-fillers and then adding a catalyst for in-situ polymerization (Fig. 3, d). The active groups of the nano-filler molecules participate in the polymerization reaction, establishing covalent bonds between the polymer and the nano-fillers (Fig. 3, c). Consequently, this graphene composite fiber does not shed ceramic or precious metal powders during use, is environmentally friendly, contains no heavy metal ions or small organic molecule additives, and provides protection such as antibacterial, anti-mite, antiviral, and UV-resistant properties.

To ensure the fiber has uniform and stable properties, it was analyzed using Raman spectroscopy, X-ray diffraction (XRD), and microscopic morphology. (1) Raman Spectroscopy: Since fiber molecules themselves have Raman characteristic peaks, directly testing the Raman spectrum of the fiber requires high microscope resolution, which is often beyond the capability of general equipment. The Raman spectrum of reduced graphene oxide in the graphene composite fiber resembles that of activated carbon and acetylene black, showing a distinct double peak structure in the 1000 ~ 2000 cm^-1^ range, but with a very small D peak for graphite. Thus, graphite can be excluded through Raman testing (Fig. 3, i). (2) X-ray Diffraction: According to the definition of graphene materials as having “no more than 10 layers,” the half-width of the graphene stacking characteristic peak (26 ~ 27°) should be greater than 2.3 ~ 2.4° to meet the definition. The XRD spectrum of the graphene composite fiber shows no obvious stacking peak at 26 ~ 27°, with a half-width much greater than 2.4°, indicating fewer than 10 layers of graphene, satisfying the definition. (3) Microscopic Morphology: Observations of the dissolved fiber samples under an atomic force microscope (AFM) revealed a significant two-dimensional flake structure with clear step characteristics when measuring height (Fig. 3, h). The single-layer thickness ranges from 2 ~ 4 nm, confirming the covalent bonding between graphene and the polymer. The AFM can also exclude granular carbon black and thicker graphite. Based on these analyses, we determined that the graphene composite fiber conforms to the characteristics of graphene materials.

To confirm the far-infrared radiation efficiency of the fiber, we commissioned the National Quality Supervision and Inspection Center for Infrared and Industrial Electric Heating Products of China to test the graphene composite fiber according to the Chinese standard GB/T 30,127 − 2013. The results showed that the far-infrared emissivity of the graphene composite fiber is 0.88 (standard ≥ 0.83), meeting the requirements of this experiment. The test report is attached (see Supplementary file 1).

### Statistical Analysis

Data are reported as mean standard deviation. Differences between the FIR-emitting outfit and placebo were assessed with a paired Student’s t-test. A *P* value of less than 0.05 is considered significant. All the statistical analyses were performed using SPSS computer software version 23 (IBM, Chicago, IL, USA).

## Results

**Fig. 4:**
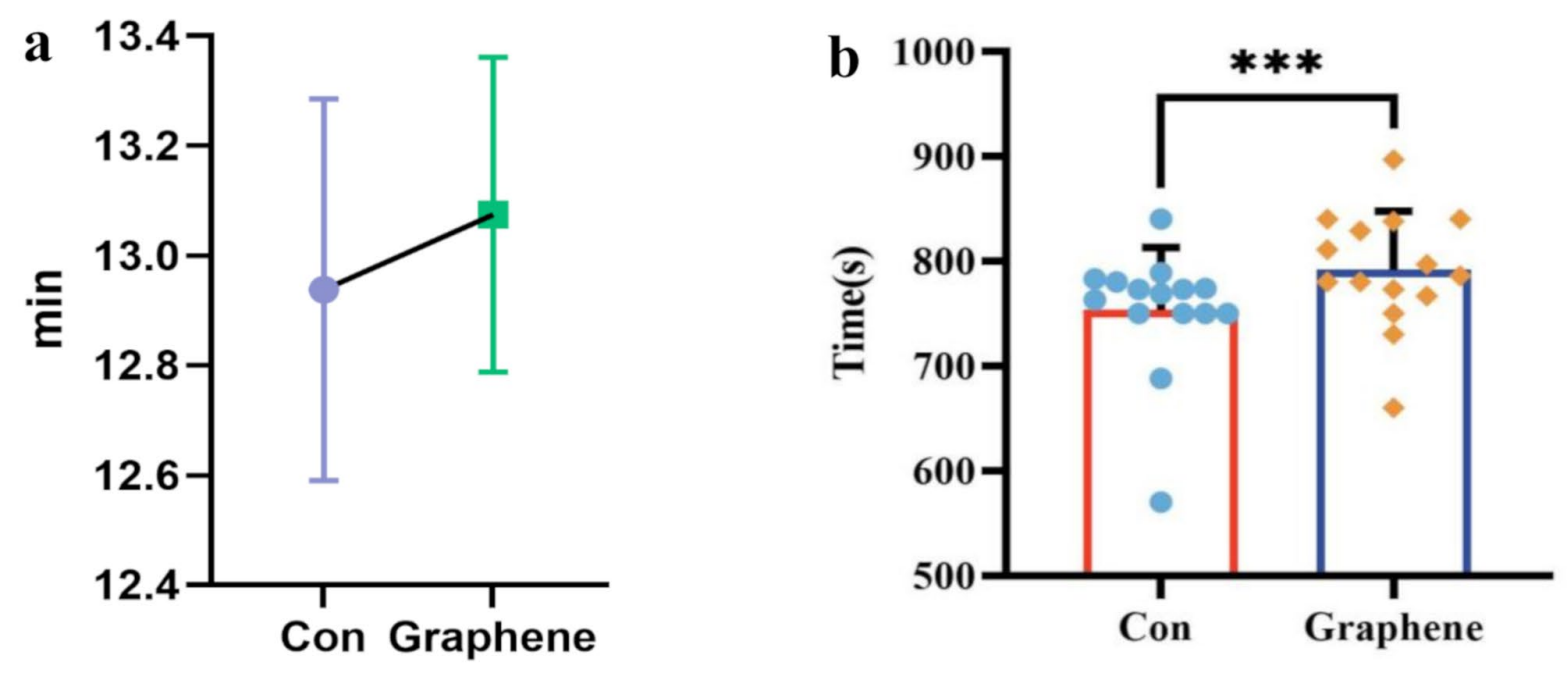
Comparison of exhaustion time between control and graphene-based FIR compression garment conditions. **a**: Mean exhaustion time presented in minutes (min). Data are shown as mean ± standard deviation (error bars). **b**: Individual and mean exhaustion times presented in seconds (s) for the control (Con) and graphene conditions. Bars represent mean values, error bars indicate standard deviations, and individual data points are overlaid. The triple asterisks (***) denote statistically significant differences between groups at *p* < 0.001

A total of 15 participants were randomly assigned in a crossover design and completed both intervention conditions. All participants were included in the final analysis for the primary outcome measures. No unintended effects or adverse events were observed or reported during the study. By comparing the data from the two maximal incremental exercise test, we found that wearing FIR garments significantly improved average maximal exercise capacity compared to placebo garments. During the incremental load-to-exhaustion test, the exhaustion time for subjects wearing graphene FIR compression garments (791.9 ± 57.6 s) was extended by 38.4 s compared to the control group (753.5 ± 54 s) (*P* < 0.001). Interestingly, using the VE/VO2—VE/VCO2 crossover method to determine the anaerobic threshold, we found that the anaerobic threshold time for the graphene FIR group (523.7 ± 98.9 s) was extended by 37.7 s compared to the control group (561.5 ± 99.1 s) (*P* < 0.001). Therefore, graphene FIR garments can significantly enhance exercise performance, particularly aerobic capacity (Fig. 4).

**Fig. 5:**
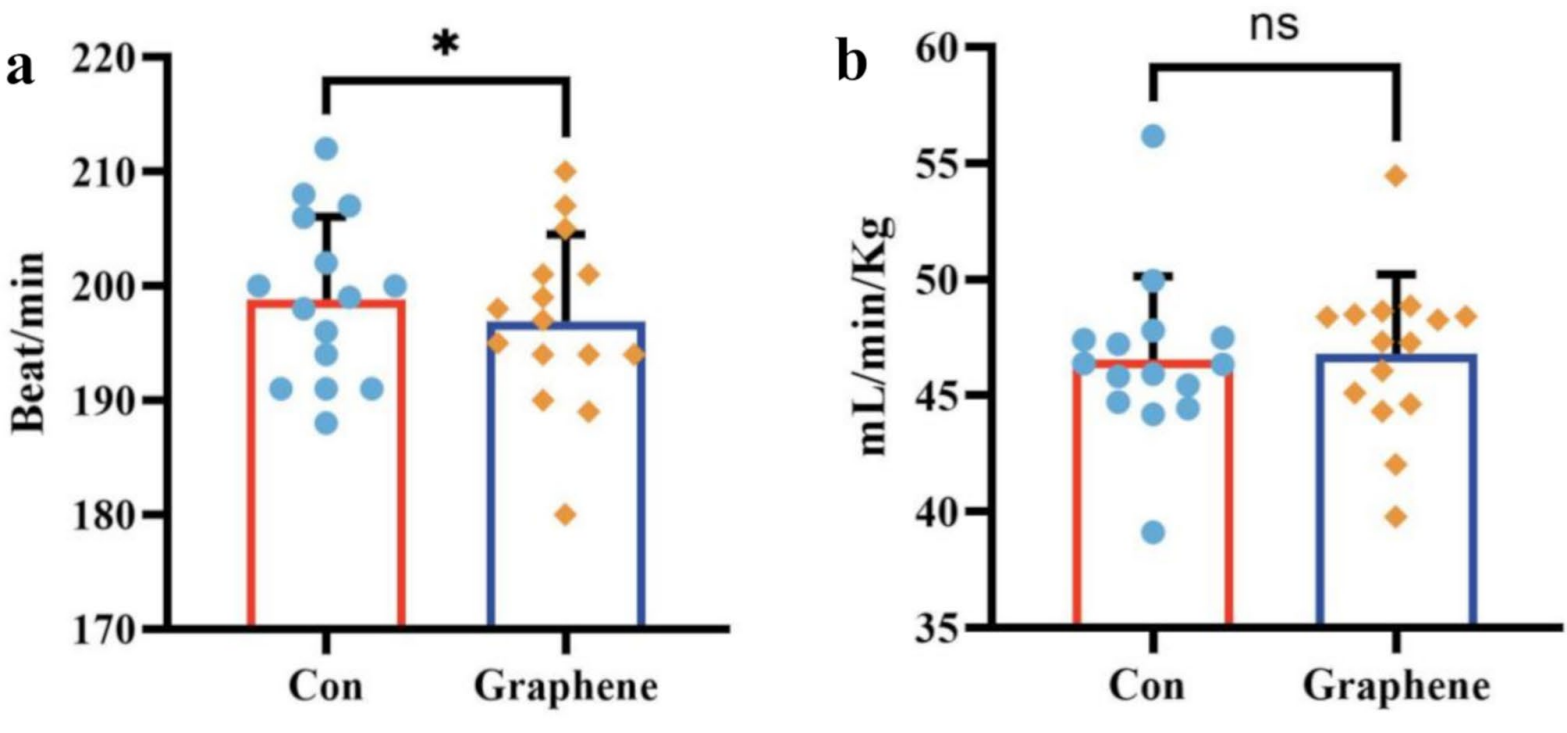
Comparison of maximal heart rate and maximal oxygen uptake (VO_2_max) between control (Con) and graphene-based FIR compression garment conditions. **a**: Peak heart rate (beats per minute, bpm). Bars indicate mean values, error bars represent standard deviations, and individual data points are displayed. The single asterisk (*) indicates a statistically significant difference between groups (*p < 0.05). **b**: Maximal oxygen uptake (VO₂max, mL/min/kg). Bars represent mean values, with error bars denoting standard deviations. Individual data points are overlaid. “ns” indicates no statistically significant difference between the two groups

**Fig. 6:**
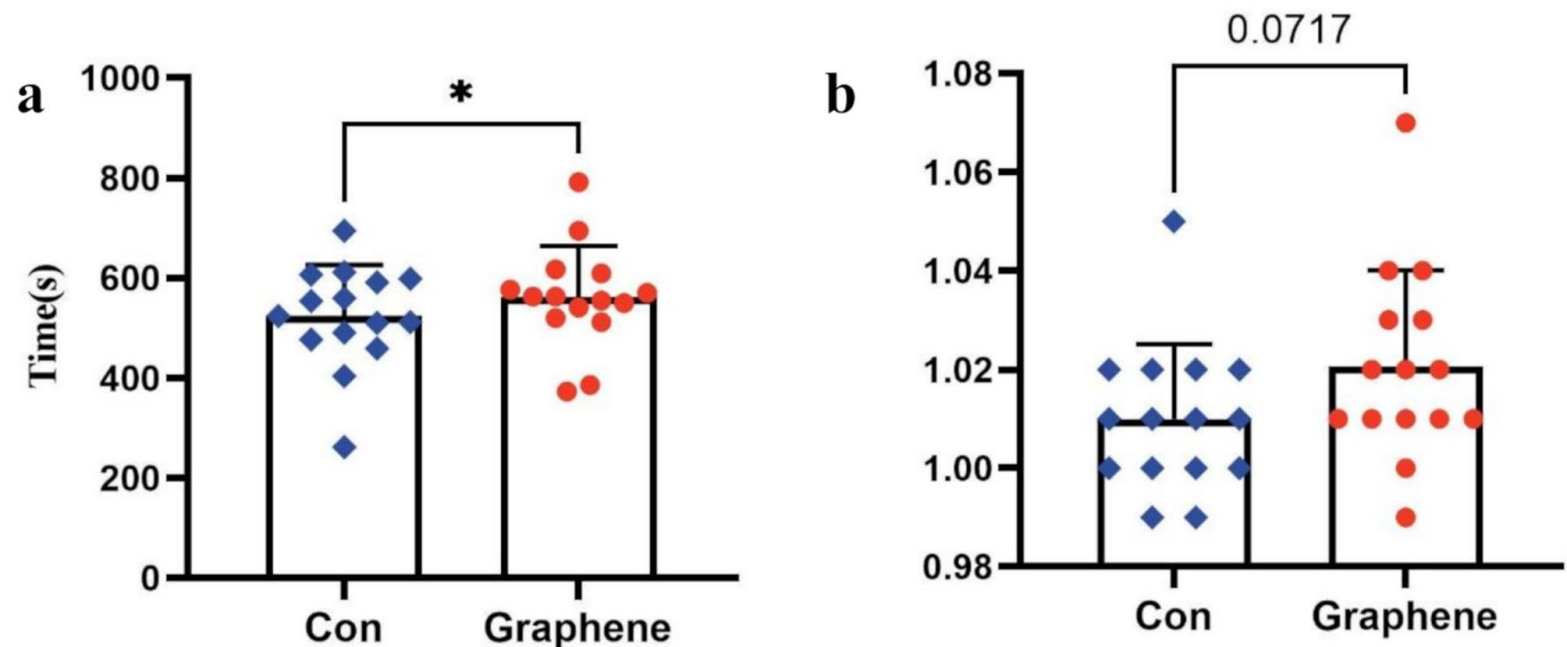
Comparison of anaerobic threshold (AT) time and respiratory quotient (RQ) at anaerobic threshold between control (Con) and graphene-based FIR compression garment conditions. **a**: Anaerobic threshold time (seconds, s). Bars indicate mean values, error bars represent standard deviations, and individual data points are displayed. The single asterisk (*) indicates a statistically significant difference between groups (*p < 0.05). **b**: Respiratory quotient (RQ) at anaerobic threshold. Bars indicate mean values, error bars represent standard deviations, and individual data points are overlaid. The exact p-value (*p* = 0.0717) indicates a non-significant statistical trend

Graphene FIR significantly reduces cardiac load during maximal exercise. During higher intensity and longer duration incremental load exercises, subjects wearing graphene FIR showed a lower maximal heart rate (Graphene group: 198.8 ± 7.8 vs. Control group: 200.3 ± 7.5, bpm) (Fig. 5). Although not statistically significant, the graphene compression garments also showed a lower respiratory quotient (RQ) (Graphene group: 1.16 ± 0.053 vs. Control group: 1.18 ± 0.052, *p* = 0.07). A lower RQ may indicate better endurance, energy efficiency, and higher aerobic capacity (Fig. 6).

Additionally, by comparing data at the same exercise intensity, defined by the earliest appearance of the anaerobic threshold in both tests, we found that the heart rate of the graphene FIR group (176.9 ± 8 bpm) was significantly lower than that of the control group (179.8 ± 7.8 bpm) (*P* < 0.05). There was no significant difference at the anaerobic threshold. However, at exhaustion, despite a difference of 38.4 s in exhaustion time, the heart rate of the graphene group (196.9 ± 7.3 bpm) was still significantly lower than that of the control group (198.9 ± 6.9 bpm) (*P* < 0.05). These results suggest that wearing graphene compression garments can significantly reduce cardiac load, improve blood circulation efficiency, and enhance overall exercise efficiency.

Although there were no statistically significant differences, some interesting data are worth noting. At rest, the average heart rate of subjects wearing FIR sportswear (71 ± 12.6 beats/min) was lower than that of the control group (73.3 ± 12.7 beats/min) (*P* = 0.06), and the oxygen consumption (7.28 ± 1.4 ml/min/kg) was also lower than that of the control group (8.06 ± 2 ml/min/kg) (*P* = 0.27). PetO2 and PetCO2 (end-tidal oxygen and carbon dioxide concentrations), which reflect arterial blood CO2 levels, were lower under maximal load conditions for the graphene FIR group (FIR: 37.2 ± 2.6 vs. Control: 37.9 ± 2.7, mmHg). The changes in respiratory quotient (RQ) are also noteworthy. At the same exercise intensity, the RQ of the graphene FIR group (0.991 ± 0.021) was slightly higher than that of the control group (0.985 ± 0.023). At the anaerobic threshold, the RQ of the graphene FIR group (1.02 ± 0.02) increased more than that of the control group (1.01 ± 0.01) (*P* = 0.07). However, at exhaustion, the RQ of the graphene FIR group (1.16 ± 0.05) was slightly lower than that of the control group (1.18 ± 0.05) (Table 2).

## Discussion

In this study, we found that graphene-based far-infrared (FIR) compression garments significantly enhanced maximal aerobic capacity and extended the duration of aerobic metabolism during incremental exercise. Wearing graphene-based FIR garments markedly reduced cardiac load during exercise, showing significant differences in both maximal heart rate and heart rate at the same exercise intensity. Additionally, resting heart rate was also lowered. These results suggest that heart rate plays a crucial role in enhancing aerobic capacity, thereby addressing the gaps in previous studies regarding the impact of FIR on heart rate variability [28, 29]. To reduce variability caused by hormonal fluctuations affecting cardiovascular and thermoregulatory responses during exercise, only male participants were included in this study. This decision limits the direct applicability of the findings to female populations. Graphene-based FIR enhances peripheral blood circulation by stimulating nitric oxide (NO) production and vasodilation, thus reducing vascular resistance. This results in improved oxygen delivery and decreased cardiac effort to maintain the same level of exercise. Consequently, heart rate at the same intensity is reduced, indicating a lowered cardiac load.

Regarding the mechanisms of FIR, Vatansever identified two main types of FIR therapies [6]. The first type, such as FIR saunas and certain electrically powered FIR generators, uses irradiance or power density sufficient to heat tissues, typically reaching tens of mW/cm². The second type, including ceramic discs, powders, and fabrics, relies on the body’s energy with lower irradiance (0.1-5 mW/cm²), and hence, does not result in tissue heating. Although the irradiance differs between these two FIR forms, their molecular and cellular mechanisms are fundamentally similar. FIR induces molecular rotation and vibration within bonds (including those in water molecules), resonating with cellular frequencies, thereby exerting its effects [30, 31]. Vatansever also suggested that FIR promotes the generation of nitric oxide (NO), which is released from mitochondrial chromophores such as cytochrome c oxidase (CCO, complex IV of the mitochondrial respiratory chain), and this NO, when bound to hemoglobin and myoglobin, leads to vasodilation [20, 32, 33]. In 2006, Yu and Chiu’s animal studies confirmed that FIR could enhance and improve skin microcirculation [9]. FIR stimulates the synthesis of NO in vascular endothelial cells, and the NO released by these cells, regulated by eNOS, mediates endothelium-dependent vasodilation by diffusing soluble guanylate cyclase, activating kinase transduction chains in arterial smooth muscle cells, inducing muscle relaxation, which consequently dilates blood vessels, reduces blood flow resistance, and thereby increases local perfusion [34, 35]. This biological effect helps enhance the oxygen transport capacity of local tissues, particularly during high-intensity exercise, reducing fatigue caused by insufficient oxygen supply [36, 37].

Mitochondria, as the cell’s energy factories, play a critical role in optimizing exercise performance. Nunes and colleagues suggested that the heat and radiation emitted by FIR materials affect membrane potential and mitochondrial metabolism [18, 38]. Vatansever indicated that the small amount of vibrational energy transmitted by non-heating FIR can open mitochondrial ion channels, particularly calcium channels, thereby increasing mitochondrial respiration. The specific mechanisms include enhanced ATP production, increased mitochondrial membrane potential, and a temporary surge in reactive oxygen species (ROS), collectively contributing to delayed muscle fatigue during exercise [6]. Leung and his team demonstrated through cellular experiments that ceramic-based FIR effectively delays the onset of fatigue, possibly due to its antioxidant properties and prevention of metabolic acidosis in muscle fibers [7].

Beyond basic research, several in vivo and in vitro studies have also shown that FIR has significant practical applications in exercise. Leung’s research revealed that FIR can stimulate the parasympathetic nervous system, reducing resting energy expenditure and significantly lowering heart rate in stressed rats and isolated frog hearts [7]. Furlan’s study indicated that using ceramic-based FIR garments could improve runners’ average speed in a 10-kilometer race [17]. Nunes found that professional soccer players wearing ceramic-based FIR garments at night after intense training experienced a significant reduction in delayed onset muscle soreness [18]. Gordon’s study demonstrated that wearing Celliant garments (FIR fabrics containing embedded particles of quartz, silicon dioxide, and titanium dioxide) 90 min before exercise could increase skin blood flow and oxygen levels [23]. Sakugawa’s research showed that using compression socks containing FIR ceramic particles for local occlusion therapy could reduce symptoms and alleviate pain in patients with lower limb edema [24]. Additionally, Mourot’s study suggested that FIR garments could effectively improve postural control in both non-athletes and professional gymnasts [8]. Manoel’s experiment further showed that participants who wore FIR garments for four days before exercise had improved neuromuscular performance of the knee extensors [22].

Graphene materials, owing to their emission spectrum overlapping with that of human tissue, exhibit superior infrared transduction capabilities [13]. Yu and Hu’s research demonstrated that FIR radiation from graphene-based flexible devices effectively inhibited tumor growth and extended the survival period of tumor-bearing mice, reducing tumor growth rates by 42% and the number of pulmonary metastatic nodules by 55% [13]. Yu specifically noted that the resonance effect enhances tissue absorption of FIR emitted by graphene devices. Recently, Li and Miao’s experiments further elucidated the mechanism of graphene FIR [12]. They found that the emission spectrum of graphene FIR devices closely matches the absorption spectrum of tissues, not only enhancing core and skin surface temperature but also increasing blood flow in the quadriceps and abdominal regions. Furthermore, graphene FIR stimulates AMPK activity by activating GPR43, thereby enhancing muscle glucose uptake. In a model of microbiota dysbiosis, graphene FIR effectively restored exercise endurance by enhancing p-AMPK and GLUT4 [39–43]. These findings suggest that graphene-based FIR therapy significantly enhances exercise capacity and glucose metabolism through the gut-muscle axis via AMPK.

Graphene-based FIR garments, with their unique infrared emission mechanism, have demonstrated a remarkable potential to enhance aerobic capacity. By improving blood flow, enhancing mitochondrial function, and delaying fatigue, these garments hold immense potential for applications in both athletic performance and recovery. Consequently, graphene-based FIR garments may represent a disruptive innovation in sports equipment, particularly in providing athletes with more efficient gear. Future research should further explore the application of graphene FIR garments across various sporting contexts to provide athletes with more comprehensive support and optimization strategies. Through continued research and practical application, graphene-based FIR garments are poised to become a pivotal tool in the future of sports science, offering new technological support to elevate athletic performance and experience.

This study has several limitations that should be taken into account when interpreting the findings. First, the study employed an acute, single-session crossover design, with only 15 participants completing the trial (out of 20 initially recruited, with five dropouts), resulting in a relatively small sample size that may have reduced the statistical power to detect differences in certain outcome variables (e.g., VO₂ max). The results therefore warrant confirmation in larger cohorts. Second, the sample was restricted to healthy, recreationally active males aged 18–25 years, excluding females, older adults, and elite athletes, which limits the generalizability of the findings. Third, although a double-blind protocol with a 7-day washout period was implemented, the study did not quantify or report garment compression pressure (mmHg), thermal insulation, objective skin/core temperature, or local blood flow (e.g., via Doppler ultrasound or near-infrared spectroscopy). Consequently, mechanistic explanations—such as potential changes in nitric oxide production, peripheral blood flow, or AMPK/GLUT4 activity—remain speculative. Finally, blinding effectiveness was not formally assessed (i.e., participants were not asked to identify which garment they believed they had worn), and thus perceptual differences or expectancy effects may have acted as potential confounders. Given these limitations, the present findings should be regarded as preliminary evidence, and future research should include larger, more diverse populations (including females and individuals with different training backgrounds), as well as incorporate mechanistic assessments such as skin temperature, local blood flow, and relevant blood or muscle biomarkers to further verify and elucidate the underlying physiological effects.

## Conclusion

This study provides compelling evidence that graphene-based far-infrared (FIR) compression garments significantly enhance aerobic exercise capacity and improve overall athletic performance. By demonstrating improvements in extending the duration of aerobic metabolism during incremental exercise, these garments show promise as a valuable tool for athletes. Additionally, the reduction in cardiac load observed, including lower heart rates during both maximal effort and at the anaerobic threshold, suggests that these garments enhance cardiovascular efficiency. The unique properties of graphene, such as its superior infrared transduction capabilities and its resonance with human tissue spectra, contribute to these performance benefits. By promoting nitric oxide (NO) production, enhancing mitochondrial function, and improving blood flow, graphene-based FIR garments offer critical physiological advantages, including delayed fatigue, which are essential for both performance and recovery.

The potential of these garments to disrupt the sports equipment industry is clear, offering a more effective alternative to traditional FIR materials. As future research explores their long-term effects and broader applications across various sports, graphene-based FIR garments are poised to become a key innovation in athletic gear. In conclusion, graphene-based FIR garments represent a significant advancement in sports science, offering promising applications for enhancing athletic performance and recovery, and are likely to play a pivotal role in the future of sports technology.

**Table 2:**
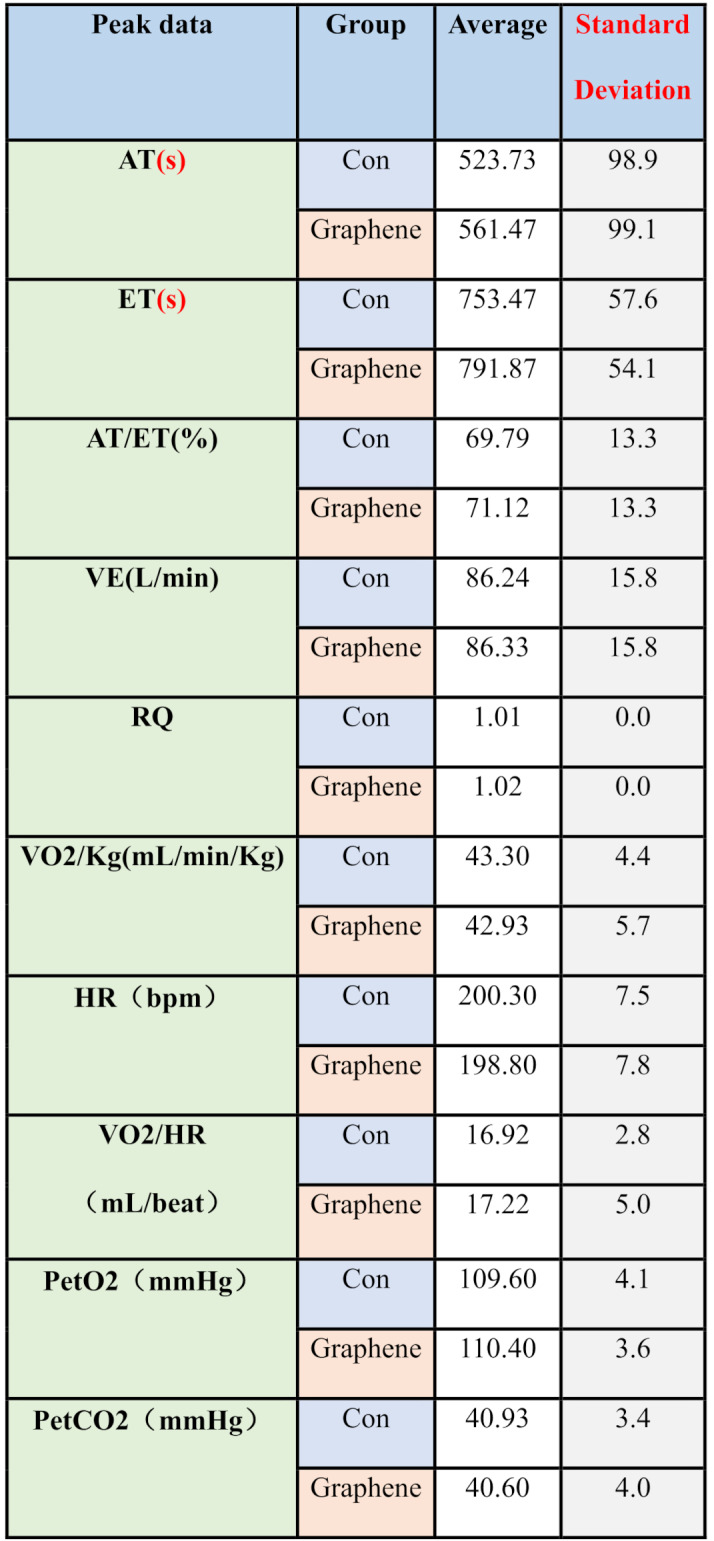
Peak Data Obtained from the Incremental Exercise Test Using the COSMED Metabolic System: Data are presented as mean ± standard deviation; *n* = 15. Comparisons between conditions were performed using paired Student’s t-tests

Abbreviations: AT, anaerobic threshold (s, seconds); Con, control; ET, endurance time (s, seconds); AT/ET (%), percentage of endurance time at anaerobic threshold; VE (L·min⁻¹), minute ventilation (liters per minute); RQ, respiratory quotient (dimensionless); VO₂/kg (mL·min⁻¹·kg⁻¹), oxygen uptake per kilogram body mass; HR (bpm), heart rate (beats per minute); VO₂/HR (mL·beat⁻¹), oxygen pulse; Pet O₂ (mmHg), end-tidal partial pressure of oxygen; Pet CO₂ (mmHg), end-tidal partial pressure of carbon dioxide. Statistical significance: *p < 0.05, **p < 0.01,*p* < 0.001

## Supplementary Information

Below is the link to the electronic supplementary material.

Supplementary Material 1: Test report

## Data Availability

The datasets generated and analyzed during the current study are available from the corresponding author on reasonable request.

## Abbreviations

AT: Anaerobic Threshold
CCO: Cytochrome C Oxidase
ET: Endurance Time
FIR: Far-Infrared Radiation
HR: Heart Rate
NO: Nitric Oxide
PetCO2: End-Tidal Partial Pressure of Carbon Dioxide
PetO2: End-Tidal Partial Pressure of Oxygen
RQ: Respiratory Quotient
SPSS: Statistical Package for the Social Sciences
VO2: Oxygen Uptake
VO2max: Maximal Oxygen Uptake
VCO2: Carbon Dioxide Output
VE: Ventilation
