# Supplementary material for: Effects of Graphene-Based Far-Infrared Compression Garments on Aerobic Capacity in Healthy Young Males: A Randomized Crossover Trial
Source: Sports Med Open. 2025 Oct 10;11:114. doi: 10.1186/s40798-025-00913-x (PMC12514096; doi:10.1186/s40798-025-00913-x)
Supplement: Supplementary file 1 — Supplementary Material 1: Test report [file 40798_2025_913_MOESM1_ESM.pdf]

181700110124 180021110348 (2018)国认监认字(040)号 中国认可 检测 TESTING CNAS L1132

检 验 报 告

TEST REPORT

检验业务号：(2019)WT-HW-01226

样品名称：纱线

国家红外及工业电热产品质量监督检验中心
China National Supervision and Test Center for Infrared and Industry Galvanothermy Product Quality

武 汉 产 品 质 量 监 督 检 验 所
Wuhan Product Quality Supervision & Inspection Institute

2019年12月25日

国家红外及工业电热产品质量监督检验中心

武汉产品质量监督检验所

检 验 报 告

检验业务号：(2019)WT-HW-01226

样品名称	纱线	规格型号	/
		商　　标	/
委托单位	杭州高烯科技有限公司	委托单位地　　址	杭州市余杭区良渚海虹新材料科技园一楼
供样单位	杭州高烯科技有限公司	供样单位地　　址	杭州市余杭区良渚海虹新材料科技园一楼
生产单位	杭州高烯科技有限公司	生产单位地　　址	杭州市余杭区良渚海虹新材料科技园一楼
抽样地点	/	检验类型	委托检验
样品数量	1筒	样品等级	/
样品基数	/	样品特性	一般
原编号或生产日期	/	样品状况	散样
送样日期	2019-12-20	送样人	张永超
注：以上样品信息由委托方提供并确认			
检验技术依　　据	GB/T 30127-2013《纺织品 远红外性能的检测和评价》		
检验结论	经检验，送检样品的远红外发射率、远红外辐照温升符合GB/T 30127-2013《纺织品 远红外性能的检测和评价》标准要求。<br>（检验报告专用章）<br>签发日期： 2019年12月25日		
备　　注	样品标注名称（商品名）：石墨烯尼龙6长纤		

批准：　　　　审核：　　　　主检：

国家红外及工业电热产品质量监督检验中心
武汉产品质量监督检验所
检 验 报 告

检验业务号：(2019)WT-HW-01226

序号	检验项目	单位	检验方法	标准要求	样本检验结果	单项评定	备注
1	远红外发射率	/	GB/T 30127-2013 中第7.1条	≥0.83	0.88	合格	/
2	远红外辐照温升	℃	GB/T 30127-2013 中第 7.2条	≥1.7	3.4	合格	/

以 下 空 白

检验员：杨一芳

国家红外及工业电热产品质量监督检验中心
武汉产品质量监督检验所
检验报告附图、附照专用表

检验业务号:(2019)WT-HW-01226

样品照片

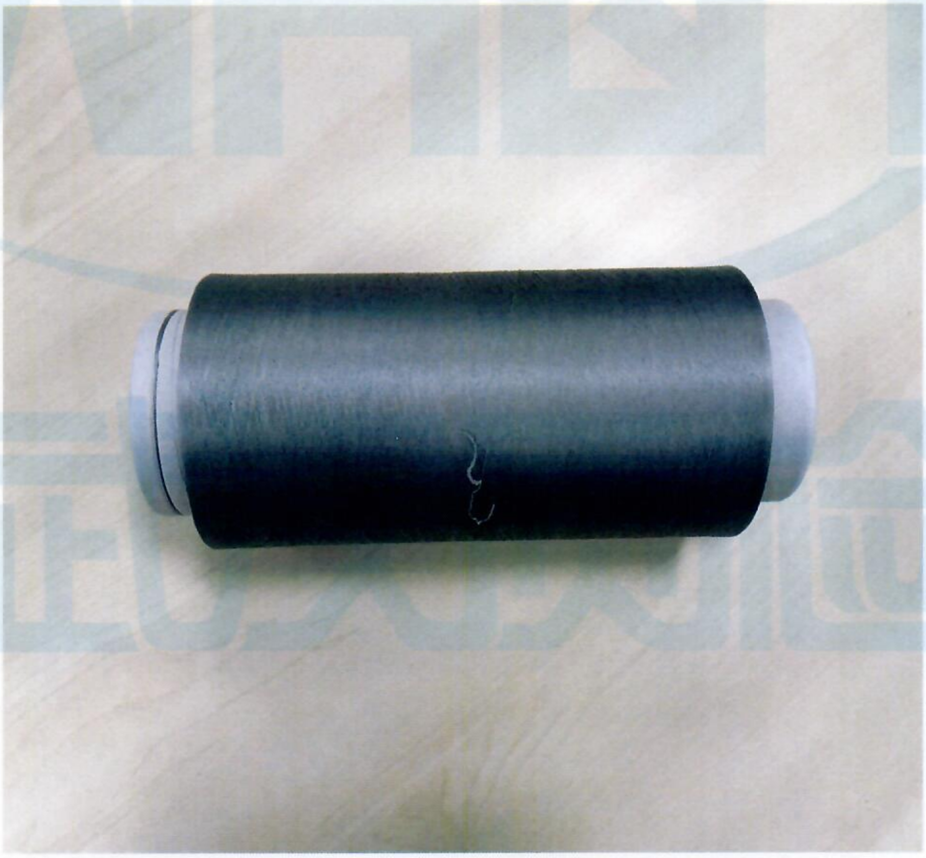

检验员：杨一芳
